# Intracortical remodelling increases in highly loaded bone after exercise cessation

**DOI:** 10.1111/joa.13969

**Published:** 2023-11-12

**Authors:** Raniere Gaia Costa da Silva, Tsim Christopher Sun, Ambika Prasad Mishra, Alan Boyde, Michael Doube, Christopher Michael Riggs

**Affiliations:** ^1^ Department of Infectious Diseases and Public Health City University of Hong Kong Kowloon Hong Kong; ^2^ Sydney School of Veterinary Science University of Sydney Camperdown New South Wales Australia; ^3^ Barts and The London School of Medicine and Dentistry Queen Mary University of London London UK; ^4^ Equine Welfare Research Foundation The Hong Kong Jockey Club Sha Tin Hong Kong

**Keywords:** bone, exercise, resorption, rest

## Abstract

Resorption within cortices of long bones removes excess mass and damaged tissue and increases during periods of reduced mechanical loading. Returning to high‐intensity exercise may place bones at risk of failure due to increased porosity caused by bone resorption. We used point‐projection X‐ray microscopy images of bone slices from highly loaded (metacarpal, tibia) and minimally loaded (rib) bones from 12 racehorses, 6 that died during a period of high‐intensity exercise and 6 that had a period of intense exercise followed by at least 35 days of rest prior to death, and measured intracortical canal cross‐sectional area (Ca.Ar) and number (N.Ca) to infer remodelling activity across sites and exercise groups. Large canals that are the consequence of bone resorption (Ca.Ar >0.04 mm^2^) were 1.4× to 18.7× greater in number and area in the third metacarpal bone from rested than exercised animals (*p* = 0.005–0.008), but were similar in number and area in ribs from rested and exercised animals (*p* = 0.575–0.688). An intermediate relationship was present in the tibia, and when large canals and smaller canals that result from partial bony infilling (Ca.Ar >0.002 mm^2^) were considered together. The mechanostat may override targeted remodelling during periods of high mechanical load by enhancing bone formation, reducing resorption and suppressing turnover. Both systems may work synergistically in rest periods to remove excess and damaged tissue.

## Graphical abstract

Bone resorption by osteoclasts is the first stage of bone remodelling, in which old bone is replaced with new bone cells, matrix and blood vessels. High‐intensity athletic activity places bone under high stress that may result in fatigue damage or fracture. Here we use cortical bone taken from horses in high‐intensity exercise and from horses in a rest period following high‐intensity exercise, from locations that are common sites of fracture, using the tenth rib as a low‐load control. We show using high‐resolution digital radiography that resorption is much more active in bones from resting than exercising horses and localises to bone regions that are susceptible to fracture. This result indicates that high‐intensity exercise exerts a site‐specific effect on suppressing targeted remodelling, which rebounds when the athletic activity stops.

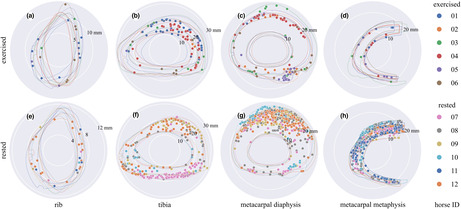

## 1 INTRODUCTION

Osteoclastic resorption of bone serves several critical functions. It is essential in the mechanism for expression of skeletal adaptation to load: resorption also facilitates the reduction of bone mass as a response to reduced loads on the skeleton, moulds the surfaces of bones to refine their geometric properties in response to altered strain (Andreasen et al., 2020; Bakalova et al., 2018; Ireland et al., 2014; Shaw & Ryan, 2012; Shaw & Stock, 2009) and is the first stage in the process to replace bone with one microstructure (Busse et al., 2010) or fibre orientation (Goldman et al., 2003, 2014; McFarlin et al., 2008; Riggs, Lanyon, & Boyde, 1993a; Riggs, Vaughan, et al., 1993b) with that of another (Collins et al., 2020). It also facilitates the repair of bone tissue by removing damaged or dead bone matrix to make way for its replacement with healthy tissue (Mori & Burr, 1993). Furthermore, resorption of bone is important for mineral homeostasis, providing a mechanism to release mineral ions bound in bone into circulation (Cappariello et al., 2014).

While osteoclastic resorption is an ongoing process in the bones of most mammals some evidence supports focal targeting of resorption in bones of the appendicular skeleton in regions where the matrix has suffered fatigue as a consequence of mechanical overload or repetitive stress injury (Martin, 2007; Plotkin, 2014). Non‐targeted or stochastic remodelling may occur as a homeostatic mechanism that has evolved to maintain the material health of the skeleton by reducing the effects of fatigue damage on bones in the long term (Parfitt, 2002). The suppression of both targeted and stochastic remodelling by experimental administration of bisphosphonates provides support for this hypothesis (Li et al., 2001). Resorption, the essential first stage of the remodelling process in dense bone, creates additional porosity that may relate to higher strain energy density (Bakalova et al., 2018), decreased elastic modulus (Gibson et al., 2006) and diminished toughness (Bell et al., 1999; Schaffler & Burr, 1988; Yeni et al., 1997).

The loss of strength and stiffness caused by increased porosity may predispose bone to accelerated fatigue or monotonic failure in the face of ongoing cyclical loads (Frank et al., 2002; Seref‐Ferlengez et al., 2015). Bone must strike a balance between the risks of tissue deterioration occurring through the accumulation of damage with the risks of failure due to increased porosity incurred during remodelling. Previous studies indicate that the balance falls in favour of allowing damage to accumulate (Seref‐Ferlengez et al., 2015) rather than attempting repair by remodelling while high loads continue.

The horse is a unique model species in which to study bone dynamics. Horse bones are large and support plentiful secondary osteonal remodelling (Boyde, 2003; Firth et al., 2005; Riggs, Lanyon, & Boyde, 1993a; Shahar et al., 2011; Stover et al., 1992), which does not normally occur in laboratory rodents or other mammals smaller than about 2 kg body mass (Felder et al., 2017). Material for detailed histological study is available post‐mortem from equine athletes with greater regularity and control than from human athletes. Histological examination of bone from horses used in exercise training tests (Firth et al., 2005) and from post‐mortem specimens from racehorses (Whitton et al., 2013) suggests that osteoclastic resorption in the third metacarpal bone is suppressed in horses that are consistently subjected to a high exercise workload compared to those forced to rest. A physiological control system may have evolved to favour the safest way to manage skeletal integrity in the face of ongoing mechanical loading, but evidence for this hypothesis is weak. The purpose of the present study was to develop the evidence base to better understand bone's response to periods of intense exertion and subsequent rest.

In this study, we measured bone remodelling, using the number and size of resorption canals as a proxy, in selected regions of bones from horses in active training and from horses subject to enforced rest. We used samples taken from horses for which we knew the exercise history with high confidence because all the animals resided, trained and raced within one organisation that kept records of all interventions.

## 2 MATERIALS AND METHODS

Bone was obtained post‐mortem from Thoroughbred racehorses (*n* = 12) at the Hong Kong Jockey Club (HKJC) that had been euthanised for reasons unrelated to the study (Table 1). Permission to use residual tissues is granted by prior agreement with the owners of all horses at the Hong Kong Jockey Club. Racing and training information was taken from the HKJC Racing information Database (The Hong Kong Jockey Club, 2021). Horses were assigned to one of two groups: “exercised” (*n* = 6) and “rested” (*n* = 6). The “exercised” group was restricted to horses that had been in regular training for racing and had undertaken intense exercise within 7 days of their death. “Intense exercise” was defined as a race, barrier trial (practice race), training gallop or training canter and “regular” as this level of exercise occurred on at least four occasions in the preceding 30 days. The “rested” group was restricted to horses that had previously been in race training (at which stage they had undertaken similar intense exercise to that of the “exercised” group) but that were undergoing a period of rest and had not undertaken any intense exercise for 30–120 days prior to euthanasia.

**TABLE 1:** List of horses in the study.

Group	ID	Age (years)	Intense exercise (days)	Exercise‐death interval (days)	Reason for death
Exercised	01	5	8	0	Drug anaphylaxis
	02	6	8	0	Tendon rupture
	03	6	6	3	Collapse during racing
	04	5	4	1	Colic
	05	5	6	0	Collapse during racing
	06	4	6	0	Collapse during fast work
	Mean	5.17	6.33	0.67	
	SD	0.75	1.51	1.21	
Rested	07	8	5	35	Advanced osteoarthritis
	08	6	5	91	Tendon rupture
	09	8	5	82	Chronic lameness
	10	8	4	45	Advanced osteoarthritis
	11	5	5	79	Unsuitable temperament
	12	6	6	94	Chronic lameness
	Mean	6.83	5.00	71.0	
	SD	1.33	0.63	24.8	

**FIGURE 1:**
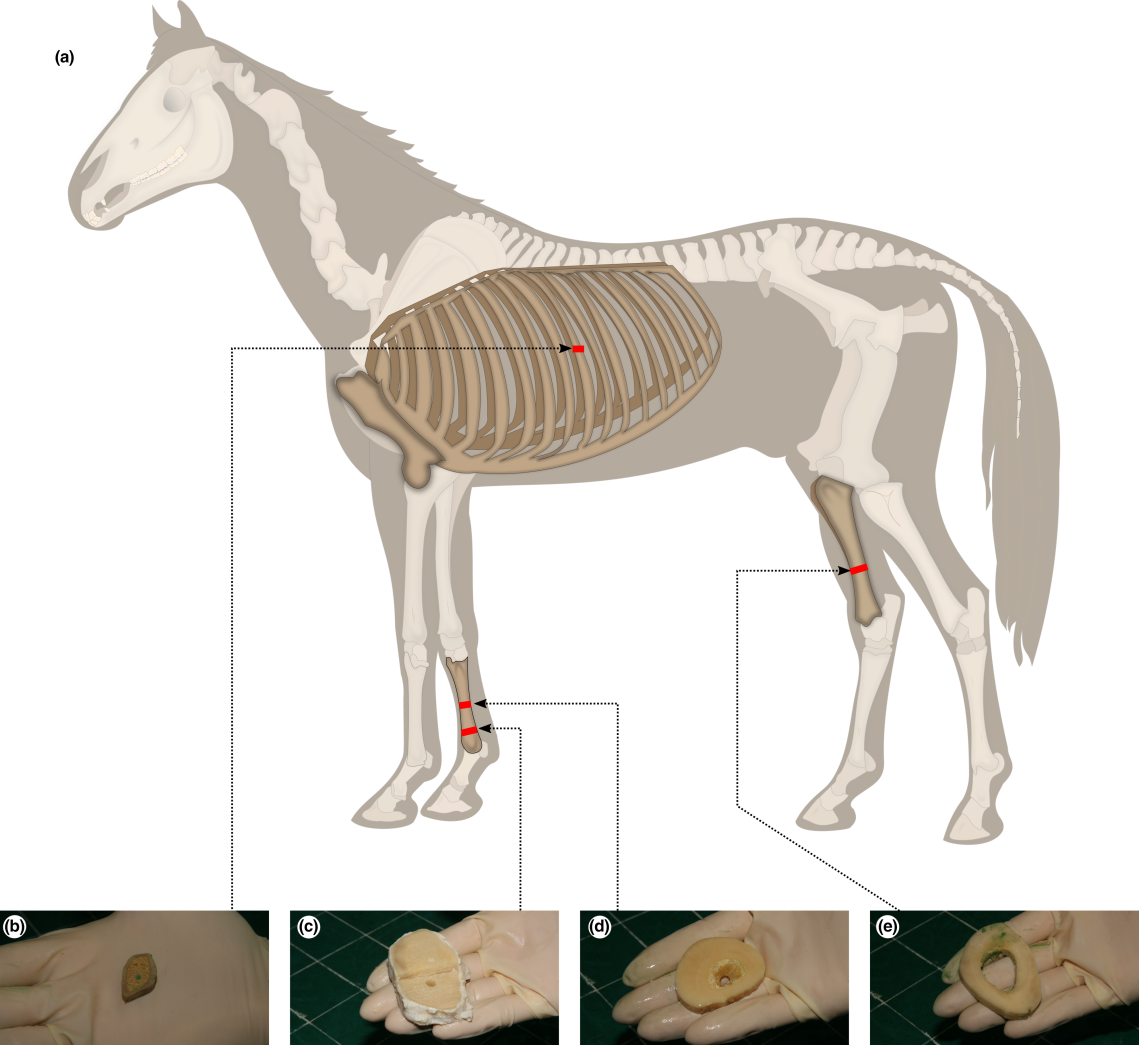
Sample collection sites mapped to (a) the whole horse: (b) mid‐diaphysis of the left tenth rib (c) lateral distal metaphysis of the right third metacarpal bone; (d) mid‐diaphysis of the right third metacarpal bone; (e) distal third region of the right tibia. This work is a derivative of “Horse anatomy.svg” by Wikipedian Prolific and Wilfredor used under Creative Commons Attribution‐Share Alike 3.0 Unported licence.

**FIGURE 2:**
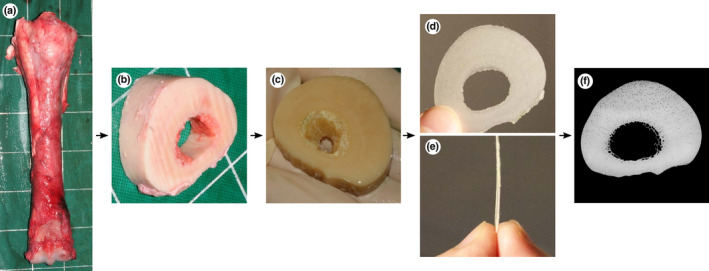
Bone processing from post‐mortem collection to microradiograph: (a) dorsal view of the right third metacarpal bone immediately post‐mortem, (b) transverse 20 mm thick slice cut with a band‐saw, (c) slice after 5 days of immersion in bacterial pronase detergent and subsequent fixation for 7 days in 70% ethanol, (d) proximodistal and (e) lateral view of 250 μm thick sections cut with an annular saw, (f) microradiograph of 250 μm thick section. Lateral to the left, dorsal to the top (c, d, f).

Bone sections were cut from the right third metacarpal bone and right tibia, at locations that are common sites for fatigue fractures in Thoroughbred racehorses (Riggs & Pilsworth, 2014). Sections were also cut from the mid‐body of the tenth left rib to represent bone of the axial skeleton that is subject to loads that increase significantly less than loads on the limbs during locomotion (Figure 1). The bones were dissected in their entirety immediately post‐mortem. Transverse blocks of 20 mm thickness were cut from the specified region of each bone using a band saw (ST‐WBS 180). The blocks from the rib, tibia and mid‐diaphysis of third metacarpal included the entire cross‐section of the bone. The blocks of the distal metaphysis of the third metacarpal included only the lateral half of the entire cross‐section. Blocks were dissected free of soft tissue including fat and periosteum before being placed in a bacterial pronase detergent (Tergazyme 10% alkaline pronase detergent, Alconox Inc, NY, USA) at 37°C for 5 days to digest remaining soft tissue. Blocks were then fixed in 70% ethanol for 7 days. Plane‐parallel sections 250 and 100 μm thick were subsequently cut from each block in a plane at right angles to the long axis of the bone using an annular saw (Leica Microsystems SP1600). Sections were stored in glycerol (Figure 2). The blocks of the distal third region of the right tibia and of the mid‐diaphysis of the right third metacarpal bone of horse 11 were severely damaged during cutting and could not be used.

**FIGURE 3:**
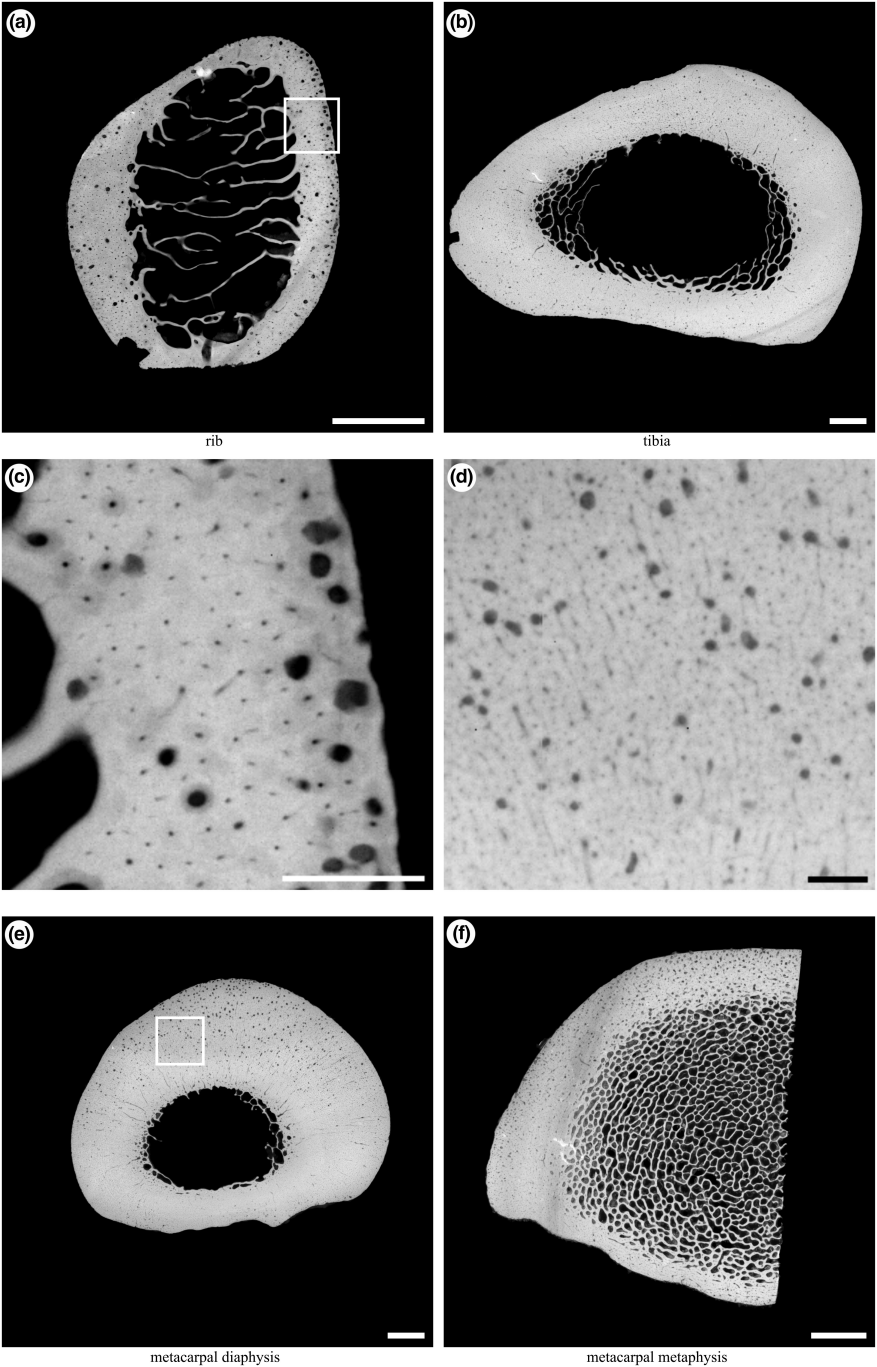
Example microradiographs obtained from each section: (a) transverse section of the mid‐diaphysis of the left tenth rib (5× magnification); (b) transverse section of the distal third region of the right tibia (2×magnification); (e) transverse section of the mid‐diaphysis of the right third metacarpal (2× magnification); (f) transverse section of the lateral half of the distal metaphysis of the right third metacarpal (3× magnification). Box in (a) magnified in (c); box in (e) magnified in (d). Scale bars: a, b, e, f, 5 mm; c, d, 1 mm. Lateral to left, cranial/dorsal to top.

Microradiographs (Figure 3) were obtained in 16‐bit DICOM format from each section by point projection digital X‐ray microscopy (Faxitron, QADOS, Sandhurst, Berkshire, UK) at 26 kV and the maximum magnification possible so that each section was captured in its entirety by a single image (Table 2, Figure 3). Images were converted to 8‐bit TIFFs for subsequent analysis, and these are available from figshare (see Supporting Information).

**TABLE 2:** X‐ray magnification, pixel spacing and minimum resolvable canal diameter (Ca.D) information for each sample site.

Bone section	Magnification	Pixel spacing (μm)	Minimum Ca.D (μm)
Rib	5	10.0	11.28
Tibia	2	25.0	28.21
Metacarpal diaphysis	2	25.0	28.21
Metacarpal metaphysis	3	16.7	18.84

*Note*: Magnification was the maximum that allowed the entire specimen to be imaged in a single frame.

**FIGURE 4:**
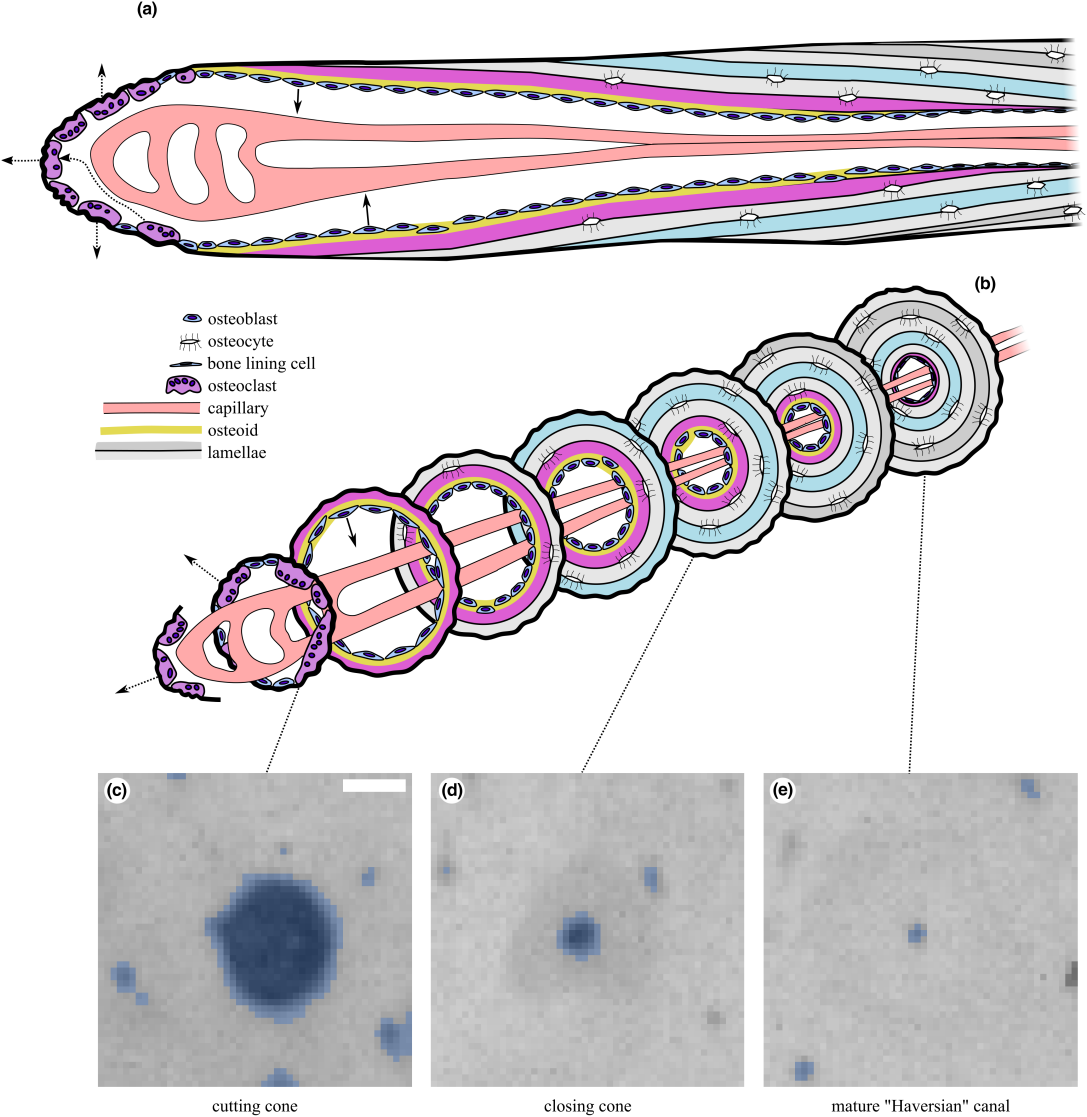
Illustration of secondary osteonal remodelling “basic multicellular unit” in cortical bone in (a) longitudinal section and (b) a series of transverse sections along its length. Representative microradiographs of the (c) cutting cone, (d) closing cone and (e) mature Haversian canal, shown with binary masks derived from processing with Fiji's Analyze Particles overlaid in blue. In this study, we classify canals into large (Ca.Ar >0.04 mm^2^; (c)) and combined large and small (Ca.Ar >0.002 mm^2^; (c + d)) and ignore the smallest canals (Ca.Ar ≤0.002 mm^2^; (e)). Scale bar 100 μm. Modified from (Doube, 2022) under CC‐BY licence terms.

Microradiographs of each section were analysed using Fiji 1.53c (Rueden et al., 2017; Schindelin et al., 2012; Schneider et al., 2012) to quantify nonmineralised areas of the section (porosities) that appear as small radiolucent spots surrounded by radiopaque cortical bone matrix. Cortical bone was segmented by manually drawing along the endosteal and periosteal boundaries. A triangle method threshold (Zack et al., 1977) was calculated for each image and applied to the greyscale microradiographs, to assign each pixel to one of “bone” or “pore” binary phases. ImageJ's Analyze Particles function was used to identify individual pores (Figure 4c–e). Particle measurements were filtered using Python to retain those with circularity between 0.3 and 1.0 and area >0.002 mm^2^. A Jupyter notebook containing the complete annotated analysis script is available at https://doi.org/10.6084/m9.figshare.19418933.v1. Size categories were selected to coincide with diameters associated with mature Haversian canals (Ca.Dm, 20–30 μm in the horse) and varying stages of resorption/infilling of resorption canals (Felder et al., 2017). The smallest particles with canal area (Ca.Ar) ≤ 0.002 mm^2^ were assumed to represent mature Haversian canals and were excluded from further analysis. Large, smooth pores at the endosteal margin of the cortex in each section that reflected transition to cancellous bone were manually excluded. The remaining pores were assumed to represent regions of bone resorption with or without subsequent formation, with the most likely descriptions corresponding to osteonal canals at the cutting cone (Ca.Ar >0.04 mm^2^) and closing cone (Ca.Ar = 0.002–0.04 mm^2^) stages (Figure 4). The diameter of each canal (Ca.Dm, μm) was calculated from Ca.Ar assuming a circular cross‐section and recorded. The cut‐off values of 0.002 and 0.04 mm^2^ were calculated from canal diameters of 50 and 225 μm (0.05 and 0.225 mm) as 0.002 mm^2^ = *π* × (0.025 mm)^2^ and 0.04 mm^2^ = *π* × (0.1125 mm)^2^ using A = *πr* ^2^, where *r* is the radius (half the diameter) of the canal. The minimum canal diameter (50 μm) was twice that of the largest pixel spacing in the images (Table 2). Cortical bone area (Ct. Ar, μm^2^), count of individual canals (N.Ca, unitless), total canal area (Tt.Ca.Ar, μm^2^), canal numerical density (N.Ca/Ct.Ar, μm^−2^) and canal area fraction (Tt.Ca.Ar/Ct.Ar, unitless) were calculated for each section (Dempster et al., 2013). Differences in the measurements between exercised and rested groups were tested for using the Mann–Whitney *U* test, implemented in SciPy (SciPy 1.0 Contributors et al., 2020). Full details of the image processing and statistical analysis are available at figshare (see Supporting Information).

## 3 RESULTS

**FIGURE 5:**
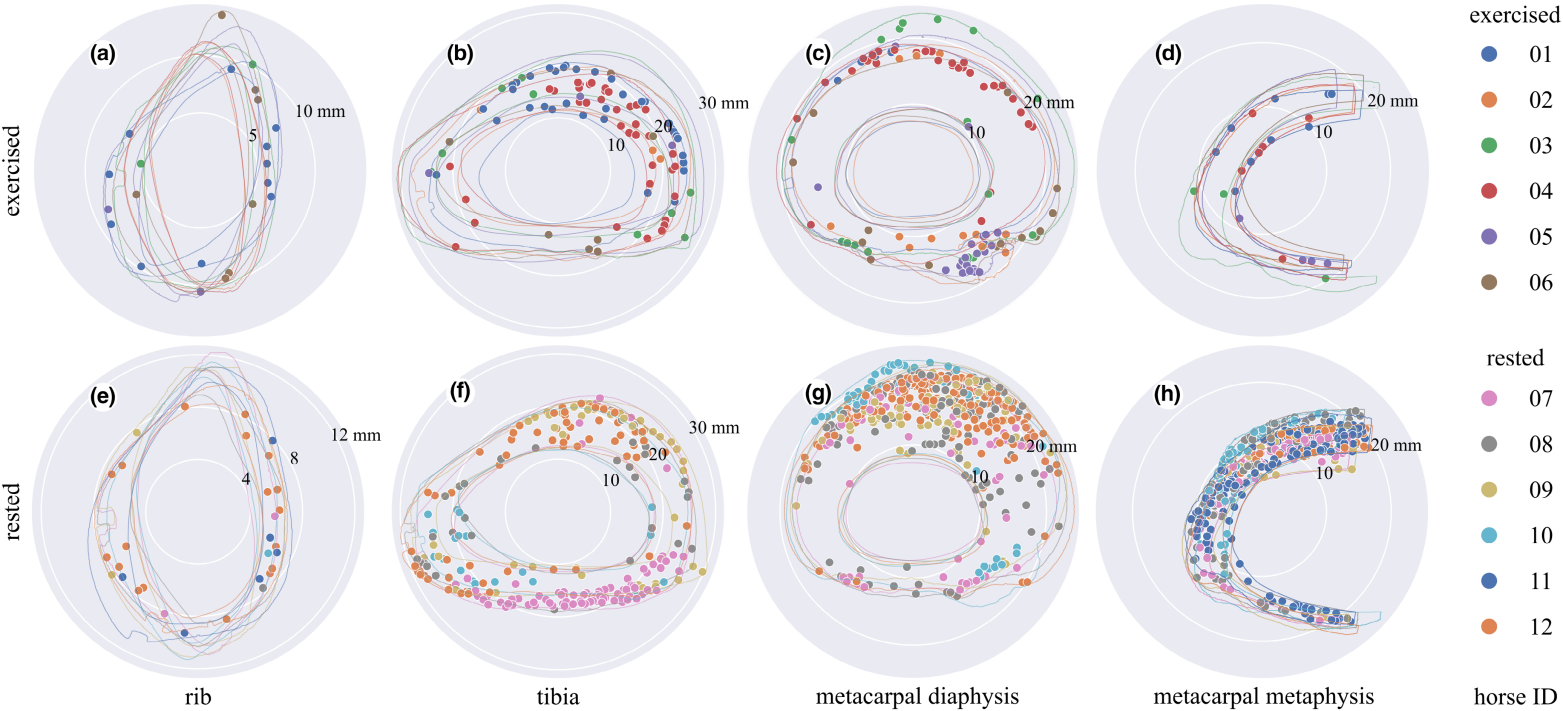
Polar plots of the largest canals representing resorption spaces or cutting cones (Ca.Ar >0.04 mm^2^) in bones from the exercised (a, b, c, d) and rested (e, f, g, h) groups, centred on each sample's centre of mass. Canals are indicated by solid dots, and periosteal and endosteal contours are indicated by outlines, all colour‐coded by horse ID. Note the larger number of large canals in the rested metacarpal and tibial sites and their anatomical distribution in the caudal tibia, dorsal metacarpal diaphysis and throughout the metacarpal metaphysis. Cranial / dorsal to the top, medial to the right. Scale in mm given by numbers indicating the radius of the white circles on each plot. See Tables 5 and 6 for statistical summaries and comparisons.

**FIGURE 6:**
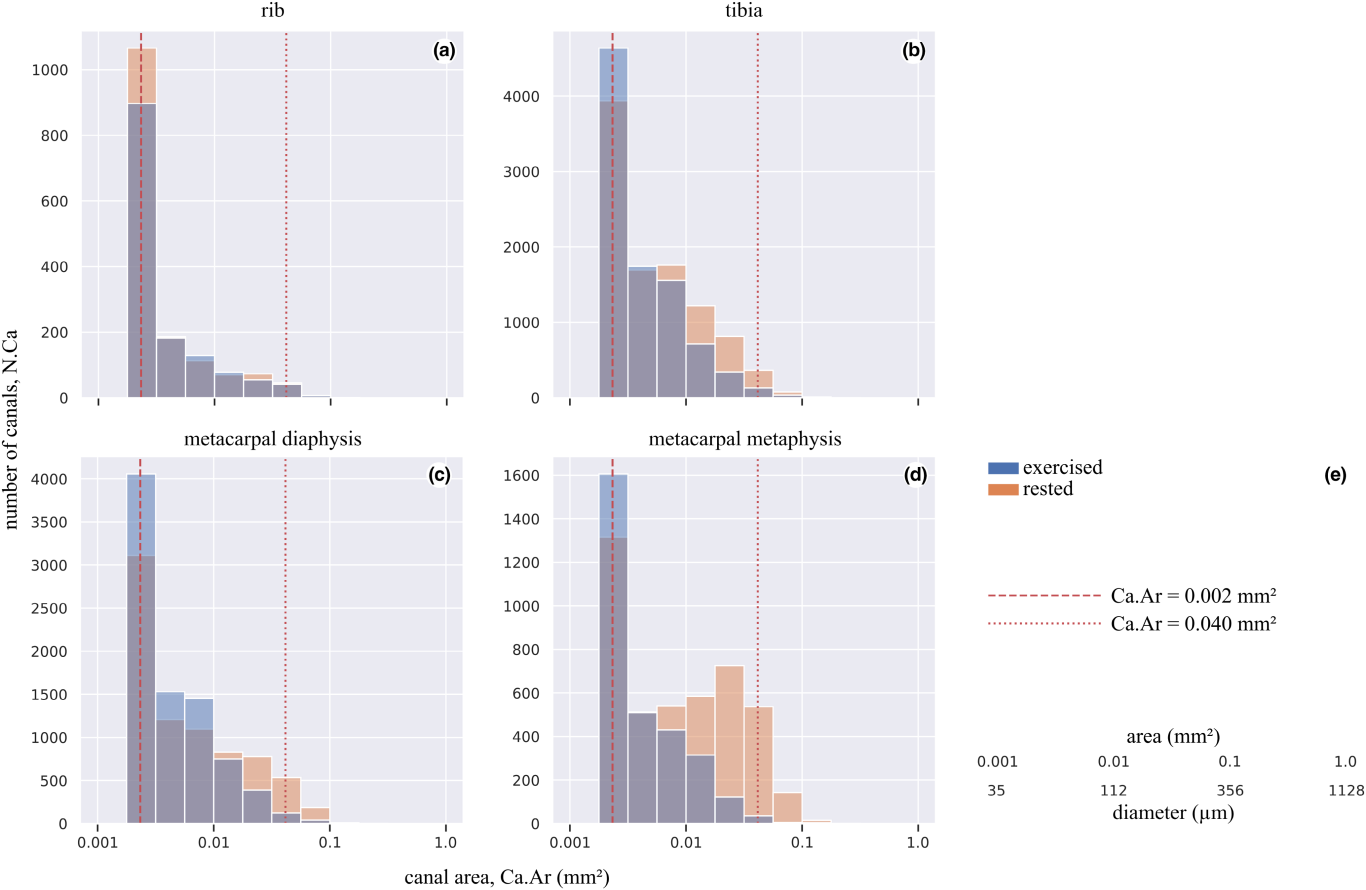
Canal number (N.Ca) summed over all samples within exercise and rested groups versus canal area (Ca.Ar) for canals with Ca.Ar >0.002 mm^2^, for each sample site. Note the greater number of large canals in the rested metacarpal metaphysis (d) and the similar distributions of canal sizes in rested and exercised ribs (a). See Tables 3, 4, 5, 6 for statistical summaries and comparisons. Vertical dashed line at 0.04 mm^2^ indicates the cut‐off value between large (resorption spaces; illustrated in Figure 5) and small (partially infilled) canals. Vertical dashed line at 0.002 mm^2^ indicates the cut‐off value between completely infilled mature Haversian canals and closing cones. Conversion between area and diameter assuming a circle provided in (e).

Where groups differed, samples from the rested group contained more canals than samples from the exercised group (Figures 5, 6; Tables 3, 4, 5, 6), which was most pronounced in the metacarpal sites and for the largest canals. Numbers of canals (N.Ca/Ct.Ar) and overall porosity (Tt.Ca.Ar/Ct.Ar) were 1.4× − 18.7× greater in rested than exercised horses' metacarpal diaphysis and metacarpal metaphysis, but were not different between rested and exercised horses in the rib or tibia (Tables 3, 5). Large canals (Ct.Ar >0.04 mm^2^) were unevenly distributed in some specimens, appearing more densely in the dorsal cortex of the rested metacarpal diaphysis (Figure 5g) and the caudal tibia (Figure 5f), while a higher number of diffusely located large canals was evident in the rested metacarpal metaphysis (Figure 5h).

**TABLE 3:** Summary of canal dimensions by site and exercise group (mean ± SD) for cutting and closing cones (Ca.Ar >0.002 mm^2^), normalised by cortical area (Ct.Ar; identical to Table 5 because they are the same specimens).

Bone	Group	Ct.Ar (mm^2^)	N.Ca	Tt.Ca.Ar (mm^2^)	N.Ca/Ct.Ar (1/mm^2^)	Tt.Ca.Ar/Ct.Ar
Rib	Exercised	80.8 ± 5.0	232 ± 102	1.36 ± 0.97	2.82 ± 1.09	0.016 ± 0.011
	Rested	79.6 ± 6.6	260 ± 130	1.49 ± 0.80	3.27 ± 1.65	0.019 ± 0.010
Tibia	Exercised	824 ± 20	1527 ± 678	9.26 ± 4.79	1.86 ± 0.82	0.011 ± 0.006
	Rested	898 ± 81	1976 ± 574	17.4 ± 4.12	2.21 ± 0.67	0.019 ± 0.004
Metacarpal diaphysis	Exercised	783 ± 73	1392 ± 561	9.06 ± 3.33	1.77 ± 0.69	0.012 ± 0.005
	Rested	816 ± 59	1552 ± 850	17.4 ± 5.80	1.92 ± 1.11	0.021 ± 0.007
Metacarpal metaphysis	Exercised	254 ± 27	505 ± 84	3.03 ± 0.86	2.03 ± 0.49	0.012 ± 0.004
	Rested	250 ± 37	730 ± 171	11.4 ± 3.52	2.91 ± 0.58	0.046 ± 0.011

**TABLE 4:** Results of Mann–Whitney comparisons between exercised and rested groups for canals with Ca.Ar >0.002 mm^2^.

Bone	Ct.Ar		N.Ca		Tt.Ca.Ar		N.Ca/Ct.Ar		Tt.Ca.Ar/Ct.Ar	
	*U*	*p*	*U*	*p*	*U*	*p*	*U*	*p*	*U*	*p*
Rib	21	0.69	15	0.69	16	0.81	17	0.94	14	0.58
Tibia	6	0.12	8	0.24	4	0.06	10	0.41	5	0.08
Metacarpal diaphysis	12	0.65	16	0.93	3	0.04	16	0.93	4	0.06
Metacarpal metaphysis	19	0.94	6	0.07	0	0.01	5	0.05	0	0.01

*Note*: *U* values tend towards 0 as the overlap between the two groups' rank distributions decreases to 0. All groups have *n* = 6 except rested tibia and rested metacarpal diaphysis which have *n* = 5.

**TABLE 5:** Summary of canal dimensions by site and exercise group (mean ± SD) for cutting cones (Ca.Ar >0.04 mm^2^), normalised by cortical area (Ct.Ar; identical to Table 3 because they are the same specimens).

Bone	Group	Ct.Ar (mm^2^)	N.Ca	Tt.Ca.Ar (mm^2^)	N.Ca/Ct.Ar (1/mm^2^)	Tt.Ca.Ar/Ct.Ar
Rib	Exercised	80.8 ± 5.0	3.67 ± 4.41	0.20 ± 0.25	0.04 ± 0.05	0.002 ± 0.003
	Rested	79.6 ± 6.6	5.00 ± 6.63	0.26 ± 0.33	0.06 ± 0.08	0.003 ± 0.004
Tibia	Exercised	824 ± 20	165.8 ± 15.8	0.92 ± 0.98	0.02 ± 0.02	0.001 ± 0.001
	Rested	898 ± 81	50.2 ± 26.0	2.98 ± 1.87	0.06 ± 0.03	0.003 ± 0.002
Metacarpal diaphysis	Exercised	783 ± 73	17.0 ± 9.25	1.06 ± 0.63	0.02 ± 0.01	0.0014 ± 0.0009
	Rested	816 ± 59	93.0 ± 44.1	5.47 ± 2.49	0.11 ± 0.05	0.007 ± 0.003
Metacarpal metaphysis	Exercised	254 ± 27	3.67 ± 3.50	0.24 ± 0.20	0.014 ± 0.014	0.0009 ± 0.0008
	Rested	250 ± 37	66.0 ± 41.6	3.89 ± 2.59	0.26 ± 0.14	0.015 ± 0.009

**TABLE 6:** Results of Mann–Whitney comparisons between exercised groups for canals with Ca.Ar >0.04 mm^2^. *U* values tend towards 0 as the overlap between the two groups' rank distributions decreases to 0. All groups have *n* = 6 except rested tibia and rested metacarpal diaphysis which have *n* = 5.

Bone	Ct.Ar		N.Ca		Tt.Ca.Ar		N.Ca/Ct.Ar		Tt.Ca.Ar/Ct.Ar	
	*U*	*p*	*U*	*p*	*U*	*p*	*U*	*p*	*U*	*p*
Rib	21	0.689	16	0.806	15	0.688	14	0.575	15	0.688
Tibia	6	0.121	4	0.055	5	0.083	4	0.055	5	0.083
Metacarpal diaphysis	12	0.648	0	0.008	0	0.008	0	0.008	0	0.008
Metacarpal metaphysis	19	0.936	0	0.005	0	0.005	0	0.005	0	0.005

## 4 DISCUSSION

We found significantly more and larger canals of a diameter that matched secondary osteonal remodelling events (i.e. the wide bore of cutting and closing cones) in the cortices of limb bones in resting horses than in horses undergoing high‐intensity exercise. It is generally accepted that large canals represent intracortical bone resorption by osteoclasts (Cooper et al., 2016; Jaworski et al., 1972); indeed, there is no known alternative mechanism for their appearance within already‐formed cortical bone. Subperiosteal cortical canals transiently appear during primary osteonal bone growth at the superficial cortical surface (Stover et al., 1992). Our finding provides further evidence to support the hypothesis that a physiological mechanism has evolved to limit or suppress resorption in the face of ongoing, high cyclical loads on bone (Dittmer & Firth, 2017; Komori, 2013), even while damage is accumulating. Because resorption is the first stage of intracortical remodelling, remodelling of fatigue‐damaged tissue may be reduced (whether targeted or not), and not increased as suggested by others (Hughes et al., 2020), while intense exercise is ongoing. Here, the rest period may have removed the suppression of resorption, allowing targeted remodelling to proceed. No relationship between osteonal remodelling and exercise group was noted in the rib, which is likely to experience only mildly elevated mechanical loads associated with increased respiratory effort at exercise.

An important function of osteoclastic resorption of bone is the repair of bone, through the replacement of damaged matrix with new, healthy tissue. Evidence that osteoclasts are recruited preferentially to target areas of matrix damage or osteocyte necrosis in cortical bone supports this concept (Martin, 2007; Parfitt, 2002; Plotkin, 2014). Accumulation of fatigue damage probably has little impact on mechanical properties of cortical bone until it becomes severe (Norman et al., 1998; Parfitt, 2002; Plotkin, 2014; Seref‐Ferlengez et al., 2015), while suppression of remodelling by half may result in three times the accumulation of fatigue damage (Allen et al., 2006; Mashiba et al., 2000). In some cases, microdamage may improve the toughness of bone (Nalla et al., 2005; Ural & Vashishth, 2007). Bone repair without resorption may occur by organic and inorganic substrates bridging and sealing cracks (Boyde, 2003; Seref‐Ferlengez et al., 2014), which might be an inherent property of mineralised tissues occurring across all vertebrate taxa (Herbst et al., 2019). In vitro mechanical testing of bone specimens machined from the third metacarpal of Thoroughbred racehorses showed that the elastic modulus and yield strength of specimens were not significantly affected by prior fatigue loading equivalent to a lifetime of racing (Martin et al., 1996). Conversely, there is good evidence that cortical porosity is negatively correlated with Young's modulus (Schaffler & Burr, 1988), compressive ultimate stress (Behrens et al., 1974) and fracture toughness (Ural & Vashishth, 2007). Changes in porosity account for more than 75% of the variability in the strength of cortical bone (McCalden et al., 1993), and fatigue‐induced damage located near cortical pores may be more likely to lead to fracture than that located in regions of high mineral content (Turnbull et al., 2014).

In the present study, resorption was determined by mapping porosities with a diameter greater than 50 μm (area greater than 0.002 mm^2^). Porosities in this size range were assumed to represent resorption spaces and partially infilled canals, whose number and size may be an accurate histological measurement of bone remodelling (Frost, 1969; Parfitt, 2002). Porosities within cortical bone are typically present in the form of osteocyte lacunae (<5 μm diameter) with associated, tortuous interconnected canaliculi (<1 μm diameter); Haversian canals at the centre of osteons (c. 20 μm diameter); Volkmann's canals, running roughly perpendicular to Haversian canals; resorption canals, in varying stages of being infilled with new bone (diameter between 20 and 400 μm). Resorption spaces in cortical bone may be the classical cutting cone passing through bone matrix or resorption occurring on the surface of existing osteonal canals (Andreasen et al., 2018, 2020). Haversian and resorption canals are roughly circular to ellipsoidal in cross‐section. The former two predominantly lie parallel with the long axis of the bone and so appear circular in transverse section of long bones. Conversely, Volkmann's canals appear as tubular or elongated oval structures, depending on their precise orientation relative to the plane of section. Larger (>600 μm wide), irregular porosities are present close to the endosteal surface of long bones, representing a transition to trabecular bone (Andreasen et al., 2020): we manually excluded these during segmentation.

Subperiosteal primary osteonal bone growth may occur and osteonal canals rapidly infill early in training in a site‐specific manner (Firth et al., 2005), conforming to the mechanostat model whereby increased strains lead to net bone deposition (Frost, 2003). Our data show decreased resorption (fewer large canals) during exercise and increased resorption (more large canals) during rest following exercise, in bone regions such as the dorsal metacarpal diaphysis that are known to experience high strains and strain rates that increase with increasing locomotor speed (Davies, 2005, 2006). Some resorption, indicated by a small number of large canals, may occur even in the most highly loaded regions during exercise periods (Figure 5), consistent with previous findings (Whitton et al., 2013).

Complete fracture of bones of the appendicular skeleton as a consequence of extension of stress fractures is relatively common in racehorses and represents a serious threat to horse welfare (Carrier et al., 1998; Riggs et al., 1999; Samol et al., 2021; Vallance et al., 2013). Better understanding of the natural biology of bone, especially in relation to repair of microdamage and how the mechanical integrity of the bone may be undermined by the repair process, is necessary in order to develop management strategies to mitigate risk. The high number of large canals observed in the rested group has important implications for physical activity management (Armstrong et al., 2004; Hughes et al., 2014; Jacobs et al., 2014; Wik et al., 2020), in particular the re‐introduction of training after returning from a prolonged rest period. High porosity may be related to reduced bone yield stress and stiffness (McCalden et al., 1997; Schaffler & Burr, 1988; Wachter et al., 2002) and may also create stress risers allowing fracture propagation (Hernandez & Keaveny, 2006). Physical readiness training, reduced marching, exercise on flat rather than hilly terrain and adhering to an exercise protocol reduce stress fractures of the lower limb bones in human military recruits (Chalupa et al., 2016; Milgrom & Finestone, 2017).

There is epidemiological evidence that racehorses returning after an 8‐week rest period are at greater risk of sustaining complete fractures of the humerus, scapula and tibia (Carrier et al., 1998; Samol et al., 2021; Vallance et al., 2013). It remains to be seen whether a gradual programme of exercise reintroduction is more bone‐safe. The time taken for completion of bone remodelling is unknown; however, osteoclasts proceed longitudinally through bone at a rate of about 40 μm d^−1^ (Jaworski & Lok, 1972), while osteoblastic infilling starts at the same time radial resorption ends (Lassen et al., 2017) and at 1.0–1.5 μm d^−1^ (Boyde & Firth, 2005; Firth et al., 2005) should take 60–90 days to infill from cement line (c. 100 μm radius) to finished canal (c. 10 μm radius) (Felder et al., 2017). Completion of remodelling at the organ scale relies on a number of secondary osteons working asynchronously in different locations and is likely to be complicated by individual variations in bone loading, training and resting patterns. Gentle preconditioning exercise that stimulates infilling and that does not suppress targeted remodelling is desirable, but the specific training regimens required to achieve this remain obscure.

Healed fractures are very common among mammals having appeared early in vertebrate evolution (Herbst et al., 2019), suggesting a survival advantage to risking fracture events because fractured bones have the capacity to heal well. We speculate that it may be a lesser cost to the organism during chronic high load periods to accumulate fatigue and defer remodelling with the concomitant fatigue fracture risk than to incur the high metabolic cost of tissue turnover and repair.

While the most plausible biological explanation for the increased number and size of canals is associated with the remodelling process that occurs with low levels of mechanical loading, the association between duration of rest period and age is unknown. We selected a rest period of 4–16 weeks based on results of previous studies (Burr, Schaffler, Yang, Lukoschek, et al., 1989a; Burr, Schaffler, Yang, Wu, et al., 1989b; McCarthy & Jeffcott, 1988) in which bone resorption was detected between 4 and 16 weeks.

To the authors' knowledge, no previous studies have reported on the differences in fracture‐free bone resorption between exercised and rested racehorses. Choosing non‐fractured horses was paramount as horses with diagnosed catastrophic or non‐catastrophic fractures may have had altered normal bone loading patterns and would not be truly representative of bone loading and resorption, although it was possible that other non‐fracture related reasons may also have caused significant lameness that may have reduced the intensity of bone loading during high‐intensity exercise. While these conditions may have influenced the normal full mobility of the horses in retirement, or even before they retired, none involved pathology that directly involved the regions of the bones studied. Furthermore, the severity of disease would not have been such as to prevent the horse from at least walking freely. The selection criteria resulted in a small sample size of 12 horses, which limits the power of the study to detect small differences between groups, and increased the possibility of random error. Matching for age was not considered practical and would have further reduced case numbers; failure to do so here resulted in the exercised horses being on average younger than the rested horses (Table 1; Holmes et al., 2014). We considered that restricting the selection criterion to high‐intensity exercise was appropriate because it has the advantage of reducing the confounding influence of training variation between horses and is consistent with the design of other studies in the field (Holmes et al., 2014; Whitton et al., 2010, 2013). The loss of a tibia specimen was unfortunate because the two‐ to three‐fold difference in N.Ca/Ct.Ar and Tt.Ca.Ar/Ct. Ar for cutting cone‐sized canals in the tibial site cannot be considered a robust result and is merely suggestive.

It was not possible to cut 100 μm sections from the entire cross‐section of the distal metaphysis of the third metacarpal bone, and so our analysis was restricted to the lateral half of the bone in this location. The palmar/palmarolateral cortex of the third metacarpal bone is the most common location for stress fractures in the distal metaphyseal region of this bone (Shan et al., 2022), and, therefore, we believe that the results from this location are likely to reflect relevant findings. The anatomical location from which specimens from the tibia were sampled was chosen because this is a common location for stress fractures to occur in our population of horses based on diagnosis by nuclear scintigraphy. While others have reported the same finding in other jurisdictions (O'Sullivan & Lumsden, 2003), it is noteworthy that complete fracture of the tibia due to stress fracture is much more common in the proximal tibia (Samol et al., 2021). Consequently, intra‐cortical resorption may be much more intense in bone of the proximal tibia than we determined from this study.

The numerical cut‐offs that we used to classify canals into large and small groups are a simplification that aids in interpretation. The microradiographic data presented here do not capture the physiological status of each canal, only their sizes. We are also aware that a hard cut‐off does not necessarily represent the diversity of modes by which resorption and infilling may occur and their dynamic nature, including substantial resting periods during which neither resorption nor deposition occur. We believe that it is helpful to separate the very largest canals (Ca.Ar >0.04 mm^2^; resorption space, cutting cone) from the moderately sized (Ca.Ar >0.002 mm^2^; infilling canal, closing cone) and smallest canals (Ca.Ar <0.002 mm^2^; mature, Haversian canal) because these categories give some insight into the overall status of the bone and in particular whether porosity that can have resulted only from resorption is more or less common depending on the exercise or rested status of the animal.

Variation in bone geometry was minimised by cutting bone blocks in pre‐determined standardised locations and visualising canals relative to the bone section's centre of mass (Figure 5). The relationship between bone remodelling and bone geometry was not within the scope of this study but is likely to be affected by differences in mechanical loading (Davies, 2006; Nunamaker et al., 1989; Piotrowski et al., 1983) and changes to the second moment of area (Merritt & Davies, 2010; Nunamaker et al., 1990) to minimise peak bone strains. It is therefore not unreasonable to assume that different training regimens may have influenced the geometry of some of the bone sections, and the relationship between bone resorption and bone geometry should be explored in future studies. The counting of canals was automated with Fiji software which should have reduced bias. A thresholding technique was used for porosity segmentation but did contend with substantial variation in background intensity due to uneven sample thickness. Given the aim of this study, two‐dimensional point‐projection X‐ray microscopy was considered a suitable imaging technique to identify bone resorption. However, future studies would better use three‐dimensional imaging techniques such as X‐ray microtomography and incorporate other markers of bone remodelling such as intra‐vital calcein labelling to evaluate bone formation rate (Boyde & Firth, 2005).

## 5 CONCLUSION

We found in limb bones, but not in ribs, that total canal area and the number of large canals, representing resorption activity, were greater in rested than in exercised animals and that large canals were concentrated in the dorsal metacarpus, which is known to experience high strains during peak athletic loading. We surmise that the resorption phase of bone remodelling is significantly suppressed by high‐intensity exercise and is subsequently reactivated during a rest period to repair accumulated damage in a site‐specific manner. During high‐intensity exercise periods, the balance between the mechanostat increasing bone mass and targeted remodelling increasing secondary osteonal cutting cone formation appears to tilt towards the former. Targeted remodelling and the mechanostat may work synergistically in rest periods to remove damaged and excess bone tissue. Numerous large canals might exacerbate the risk of catastrophic failure should intense exercise be reintroduced prior to the osteoblastic infilling that completes the bone remodelling cycle. Future studies should be directed at determining appropriate training regimens to protect athletes' bones when they return to exercise following an extended period of rest.

## AUTHOR CONTRIBUTIONS

Raniere Gaia Costa da Silva: formal analysis, methodology, writing original draft. Tsim Christopher Sun: methodology, writing original draft. Ambika Prasad Mishra: writing. Alan Boyde: obtained the grant for the Faxitron and performed the imaging. Michael Doube: supervision, writing original draft. Christopher Riggs: supervision, conceptualisation, methodology, writing original draft. All authors contributed to review and editing and gave final approval for publication and agreed to be held accountable for the work performed therein.

## CONFLICT OF INTEREST STATEMENT

The authors have no conflict of interest.

## Data Availability

TIFF images (8‐bit) as used for analysis are available at doi:10.6084/m9.figshare.c.5910674. Full bit‐depth (16‐bit) DICOMs are available upon reasonable request. Bone statistics are available at doi:10.6084/m9.figshare.19410431. Pore information is available at doi:10.6084/m9.figshare.19410452. Full details of the image processing and statistical analysis are available at doi:10.6084/m9.figshare.19418933.
